# The Background Structure of Entrepreneurial Team and Strategic Investment Decisions: A Collective Psychological Capital Perspective

**DOI:** 10.3389/fpsyg.2019.01416

**Published:** 2019-06-20

**Authors:** Li Xin Guo, Kun-Hwa Lu, Ya-Fang Cheng, Chi-Fang Liu

**Affiliations:** ^1^Department of Business Administration, Huaiyin Institute of Technology, Huai’an, China; ^2^Department of Business Administration, Cheng Shiu University, Kaohsiung, Taiwan

**Keywords:** collective psychological capital, functional background, background structure, investment decision, entrepreneurial team

## Abstract

Drawing from the perspective of collective psychological capital, this study analyzes the internal mechanism of how top management structure influences R&D, and marketing investment decisions. Utilizing a sample of 346 Chinese listed companies in high-tech industries from 2012 to 2017, we examine the relationship between the proportion of entrepreneurial team with technology, marketing-related background, and R&D marketing expenditure. The empirical results show the proportion of entrepreneurial team with the technological background is positively related to R&D expenditure and negatively related to marketing expenditure. On the contrary, we also find that the proportion of entrepreneurial team with a marketing background is a negative correlation with R&D expenditure and positive correlation with marketing expenditure. Our study has expanded the perspective and scope of the research on the antecedents of strategic investment decisions, and the practical implications are discussed.

## Introduction

Companies pursuing different business orientations generally have different strategic investment decisions – companies with entrepreneurial orientation often allocate more resources to R&D activities, while companies with market orientation usually allocate more resources to marketing activities. Under the limited resources, there may be a competition for resources between R&D and marketing activities. However, what determines this type of resource allocation decision of company? The existing literature has not yet provided satisfactory answers. Previous studies have extensively examined the direct and indirect effects of entrepreneurial orientation on firm performance (Wiklund and Shepherd, 2003, 2005; Moreno and Casillas, 2008; Stam and Elfring, 2008; Engelen et al., 2015), and the marketing literature also comprehensively analyzes the consequences of market orientation (Narver and Slater, 1990; Han et al., 1998; Morgan et al., 2009; Lee et al., 2015; Devece et al., 2017). In addition, some scholars have also studied the relationship between entrepreneurial orientation and market orientation (Miles and Arnold, 1991; Baker and Sinkula, 2009). However, the determinants of entrepreneurial orientation and market orientation have not been fully studied, and we still do not understand why different companies have different business orientations and resource allocation decisions.

The psychological capital literature has highlighted the important role of positive psychological state on individual attitudes, behavior, and work performance (Luthans et al., 2004, 2005, 2006, 2007a, 2008, 2010; Luthans and Youssef, 2004; Avey et al., 2009, 2010, 2011; Newman et al., 2014), and four positive psychological resources have been identified by Luthans et al. (2004): self-efficacy, hope, optimism, and resiliency (Luthans and Youssef, 2004), which are conceptualized as a higher-order construct of psychological capital (Newman et al., 2014). Some empirical studies have provided empirical evidences that individual psychological capital has a positive impact on job performance (Luthans et al., 2005, 2008, 2010), work attitudes, and behavior (Avey et al., 2010). Recently, some empirical studies have further expanded the research scope of psychological capital at the individual level and provided some interesting findings, for example, Leon-Perez et al. (2016) report that employees’ psychological capital positively influence their service quality and negatively affect their burnout. Chen et al. (2017) find that leaders’ psychological capital has positive impact on that of their followers by the mediation role of organizational identification, Hu et al. (2018) have examined the mediating role of subordinate psychological capital on the relationship between authentic leadership and proactive behavior. The psychological capital literatures at the individual-level have attracted many scholars’ interest in studying psychological capital at the group or organizational level (Hambrick and Mason, 1984; Walumbwa et al., 2011; Dawkins et al., 2015; Heled et al., 2016; Luthans and Youssef-Morgan, 2017). For example, Walumbwa et al. (2011) report that collective psychological capital of groups is significantly correlation with their group-level performance and citizenship behavior, Dawkins et al. (2015) develop a multilevel-multi-referent framework to conceptualize collective psychological capital, Heled et al. (2016) find that the team’s psychological capital is positively related to the team’s organizational citizenship behavior. Unfortunately, few scholars have noticed that the collective psychological capital of TMT may affect the company’s strategic investment decisions.

Indeed, upper echelons theory has long advocated that company strategic choices are the outcomes of TMT’s decision-making and behavior (Walumbwa et al., 2011), thus, we believe that business orientation and strategic investment decisions will be greatly influenced by TMT’s collective cognition, and psychological state. Because psychological capital is a state-like construct, it may change with individual, or group experiences and tasks, for example, when confronted with the same decision-making task, TMTs with different background structures may have different collective psychological states and different decision preference. Therefore, based on the perspective of collective psychological capital, this study attempts to analyze and test the influence of entrepreneurial team’ background structure on strategic investment decisions, this will have the following research implications.

First, our study contributes to the business orientation and strategic decision literature. As noted as above, the outcomes of entrepreneurial orientation and market orientation have been broadly analyzed and examined empirically, but the antecedents of them have not been fully investigated and tested. Although a few studies have analyzed the impact of executives’ individual characteristics on entrepreneurial orientation, such as Boling et al. (2016) examine the effect of CEO tenure on entrepreneurial orientation. Barker and Mueller (2002) investigate the influence of CEO characteristics on R&D investment. While these studies mainly focus on individual level. Our study enriches and expands the perspective and scope in this field by the analyzing group-level phenomenon.

Second, our study also contributes to the psychological capital literature. The literature of organizational behavior has extensively studied the antecedents and outcomes of individual psychological capital. However, the study at the group-level is still in its infancy, especially the research on the influencing factors of group’s psychological capital. We believe that the different background structure of senior executives will affect TMT’s collective psychological capital, especially when dealing with different decision-making tasks. Therefore, our study extends the research on psychological capital in the field of micro-organizational behavior to the field of strategic management research.

Finally, our study is also helpful to understand and reconcile the contradiction between entrepreneurial orientation and market orientation, as Matsuno et al. (2002) mentioned, there is a potential tension between entrepreneurial proclivity and market orientation, under the constraint of resources, it is difficult for a company to provide sufficient resources for both the two orientations simultaneously, both theory and practice require a clear understanding of how a company decides its R&D and marketing investment decisions. Our study contributes to this line studies by analyzing the distinctive effect of the proportion of entrepreneurial team with different background (technology-related and marketing-related background) on different strategic investment decisions (R&D and marketing investment decisions).

## Theoretical Background and Hypothesis

### Theoretical Foundation

Psychological capital at individual–level has been defined as “one’s positive psychological state of development that is characterized by (1) having confidence (self efficacy) to take on and put in the necessary effort to succeed at challenging tasks; (2) making a positive expectation (optimism) about succeeding now and in the future; (3) persevering toward goals and, when necessary, redirecting paths to goals (hope) in order to succeed; and (4) when beset by problems and adversity, sustaining and bouncing back and even beyond (resilience) to attain success” (Luthans et al., 2007b; Luthans and Youssef, 2007). Based on the works of Bandura (1997), Walumbwa et al. (2011), and Luthans et al. (2007b) further define collective psychological capital as the “group’s shared psychological state of development that is characterized by the above four attributes,” and suggest that “group’s collective psychological capital is a product of interactive/coordinative dynamics and leadership,” it can produce “desired behaviors and performance outcomes” (Walumbwa et al., 2011, p. 6-7).

We argue that the background structure of entrepreneurial team will influence business orientation and related spending decisions, the reason is that both the background-structure of entrepreneurial team and the characteristics of strategic decision-making tasks will affect the TMT’s collective psychological capital. In other words, TMT’s collective psychological capital will vary with the changes of its structure and decision-making tasks. Indeed, some empirical studies show that psychological capital is a state variable, for example, the empirical results of Luthans et al. (2007a) suggest that psychological capital is a “state-like” construct. On the other hand, prior studies have theoretically and empirically analyzed the influence of collective psychological capital on group behavior and group-level performance, for example, Walumbwa et al. (2011) report that group’s collective psychological capital has positive influence on group performance and citizenship behavior, Heled et al. (2016) find that team’s psychological capital is positively related to the team’s organizational citizenship behavior. Therefore, from the perspective of collective psychological capital, we analyze how the structure of entrepreneurial team affects the company’s business orientation and related decisions as follows:

### The Technological Background Structure of Entrepreneurial Team and Strategic Investment Decisions

Hambrick and Mason (1984) claim that entrepreneurial team are the main strategic decision makers of company, and the company’s strategic decisions and behaviors are the outcomes of the cognition and interaction of TMT members. TMT is generally composed of members with different professional and work experience backgrounds, the personal backgrounds of entrepreneurial team not only impact individual cognition, but also affects the interaction style with other team members, thus affecting the group cognition and collective interaction style, because psychological capital is a “state-like” construct (Luthans and Youssef-Morgan, 2017), the collective interaction style may influence TMT’s collective psychological capital of development, and especially when they face various strategic decision tasks that will lead the company to different growth directions. Unlike other background entrepreneurial team, entrepreneurial team with technology-related background may have stored more science and technology-related information and knowledge in their brains, because they may have experienced a great deal of science and technology related training, learning, and working. When a technological top manager is involved into technology-related strategic decision, he/she can quickly understand the prospect and importance of the project, and know how to ensure the success of the project, thus, he/she is prone to positive psychological states such as confidence, optimism and hope. When a technological top manager is being beset by problems and adversity of the project, based on his/her technology-related knowledge, he/she can quickly identify the existing problems and find out the path to success, in this situation, he/she show a higher resilience.

Therefore, we believe that entrepreneurial team with technology-related backgrounds tend to choose the strategies of entrepreneurial orientation, and support allocating more resources to technology-related activities, such as R&D and technological innovation activities, because they have higher psychological capital in the process of completing technology-related tasks. On the contrary, because technological entrepreneurial team may lack of marketing-related knowledge and work experience, they may not be able to accurately understand and grasp the current situation and future trend of the market and competition, thus, when they are involved in marketing-related strategic decisions, they cannot judge whether the relevant marketing investment can achieve corresponding returns, and it is difficult for them to generate positive psychological states such as confidence, optimism, and hope. Specially, when the marketing investment fails to bring the expected benefits to the company, they are prone to fall into pessimism, and oppose the continued allocation of resources to related marketing activities. Therefore, we argue that entrepreneurial team with technology-related backgrounds will not tend to support a strategic decision of market orientation, and may have negative influence on the investment in marketing activities.

On the other hand, when dealing with decisions related to entrepreneurship and market orientation, entrepreneurial team with technology-related backgrounds not only show different psychological capital and decision preferences at the individual level, but also at the group level. At this point, it can be explained from two aspects. First, when technological entrepreneurial team are involved in the decision process of technology-related projects, they can effectively communicate and discuss with each other because they have shared or similar knowledge and experience, for example, they can analyze the advancement and operability of technology-related investment projects, accurately understand the prospect and key successful factors of the project, and find ways to break through the technical difficulties, thus making technological top management team full of confidence and hope for the success of the project.

As a result, the interaction between technological entrepreneurial team increases the psychological capital of the group, making it easy for them to reach an agreement on technology-related investment decisions. Secondly, technological entrepreneurial team can also enhance the confidence of other background executives in technology projects by interpersonal interaction, for example, technology executives can carefully explain the prospect of technology projects to other executives, help to build other executives’ confidence on the project, and improve other executives’ psychological capital, which is conducive to persuade other executives to support the project. However, when technical executives are involved into the decision process of marketing-related investment, they may object to investing in marketing program not only because they may lack the positive psychological state to the marketing project, but also because marketing investment may reduce their preferred R&D investment under the company’s limited resources, thus they have motivation to prevent the approval of marketing investment decisions.

In China, the economic system is still in the process of continuous reform and improvement. With a high degree of environmental uncertainty, strategic decisions of listed companies are generally determined by total entrepreneurial team. In the practice of collective decision-making of the Chinese listed companies, the strategy choices are ultimately decided by voting; whether an investment decision can be approved or not, depends on the number of votes of support. As mentioned above, technical entrepreneurial team tend to support the decisions of technology-related investment; so the number of technical entrepreneurial team can be regarded as the number of votes of support.

Therefore, for the decision of technological R&D investment, when the proportion of technology executives in listed companies is relatively high, they can create higher TMT’s collective psychological capital by the individual and group interaction mechanism, and TMT may show a positive psychological state which is characterized by self-efficacy, optimism, hope and resilience to the success of R&D projects, thus increasing the possibility of approval of R&D investment proposals. In other words, the higher the proportion of entrepreneurial team with technology-related backgrounds in listed companies, the greater their influence on strategic decisions, and the higher the collective psychological capital of TMT in technology-related investment decisions, the greater the probability that listed companies will choose entrepreneurial-oriented strategies and increase the company’s R&D investment, which will reduce the company’s investment in marketing under limited resources. And vice versa, under the constraints of resources, when the proportion of technology executives in listed companies is relatively low, their influences on TMT’s collective psychological capital is relatively small, and the R&D investment proposal may not be approved, or only a discounted proposal is chosen, for example, reducing the amount of investment in R&D. Therefore, we hypothesize as follows:

Hypothesis 1: Ceteris paribus, the proportion of entrepreneurial team with technology-related background is positively related to R&D expenditure.
Hypothesis 2: Ceteris paribus, the proportion of entrepreneurial team with technology-related background is negatively related to marketing expenditure.

### The Marketing Background Structure of Entrepreneurial Team and Strategic Investment Decisions

Compared to technical entrepreneurial team, entrepreneurial team with marketing background have more market knowledge, information and marketing practice experience, they grasp more knowledge and information concerning to the customers’ demands and preferences, know how to meet these demands and create value for customers, and thus they can find effective ways to improve customer satisfaction and customer loyalty. In addition, compared with other executives, marketing background executives may more accurately identify the main competitors of company, and understand the advantages and disadvantages of competitors, thus knowing which marketing methods can effectively improve the company’s market competitiveness and market share. Therefore, marketing-background executives naturally have a preference to invest in marketing activities, when they are involved into the investment decision related to marketing, they may have more confidence on the success of marketing program, because they know the paths to preform marketing goals, they have more possibility to produce positive psychological state such as hope and optimism. When the marketing activities fail to achieve the expected results, they can effectively find the existing problems and design relevant solutions. Therefore, when the marketing background executives face the difficulties and obstacles of marketing activities, they can still show a positive psychological state such as resilience. Based on the above analysis, we believe that entrepreneurial team with marketing backgrounds may have higher psychological capital in the decision process of marketing investment, thus, they may tend to support the choice of market-oriented strategies and have a positive impact on the company’s marketing-related investment.

At the group-level, marketing-background executives can communicate with each other, share marketing-related knowledge and experience, and deeply discuss marketing decision proposal, thus, they can predict the potential problems of the proposal and prepare solutions in advance, further improving the collective psychological capital of marketing-background entrepreneurial team. In addition, marketing entrepreneurial team can also introduce to other executives the advantages of the marketing proposal, the possibility of its realization, and the benefits it can create for company, these behaviors may enhance other entrepreneurial team’ confidence in the success of the marketing proposal, and persuade other executives to support the proposal. The higher the proportion of marketing entrepreneurial team in a company, the greater the influence of marketing entrepreneurial team on the company’s strategic decisions, and the greater the collective psychological capital of TMT for the marketing proposal, the higher the probability that marketing proposal will be approved. Therefore, we believe that the proportion of entrepreneurial team with marketing background will have a positive impact on the company’s marketing investment.

On the other hand, entrepreneurial team with marketing-background may show different psychological states when they participate in the decision process related to technology-related R&D investment. First, because entrepreneurial team with marketing background generally do not have in-depth and broad technology-related knowledge and information, they may not be able to effectively evaluate and judge the success possibility of technology-related R&D projects. Therefore, they may not be able to build confidence, hope and optimism for the R&D investment project, which may lead to negative psychological states such as pessimism and suspicion. When entrepreneurial team with marketing-background have a higher proportion in TMT, their negative psychological states will influence other managers’ emotions and reduce TMT’s collective psychological capital. Secondly, under the limited resources of company, too much research and development investment may reduce the free resources of company, which may lead to a reduction in the marketing investment budget. Some marketing programs with high-risk and high-return may not be able to obtain sufficient resources to be performed. Therefore, marketing-background executives may not support the company’s R&D investment. The higher the proportion of marketing-background executives, the greater their influence on the company’s strategic decisions, and the lower the possibility of approval of R&D investment decisions. Therefore, we believe that the proportion of entrepreneurial team with marketing background will have a negative impact on the company’s research and development investment.

In short, we argue that the marketing-background structure of entrepreneurial team with will have higher individual and collective psychological capital when they are involved in marketing investment decisions, so they may have a positive influence on marketing investment. When entrepreneurial team with marketing background are involved in the decision process of R&D investment, they may have lower individual and collective psychological capital, therefore, entrepreneurial team with marketing background will have a negative impact on R&D investment. The higher the proportion of marketing-background managers in TMT, the stronger the above effect. Thus, we hypothesize as follows:

Hypothesis 3: Ceteris paribus, the proportion of entrepreneurial team with marketing-related background is positively related to marketing expenditure.
Hypothesis 4: Ceteris paribus, the proportion of entrepreneurial team with marketing-related background is negatively related to R&D expenditure.

## Materials and Methods

### Sample

To test the above hypotheses, we collected panel data of high-tech listed companies at the period 2012–2017 in China. These companies belong to the following industries: computer and communication technologies, instrument and meter manufacturing, electronic equipment manufacturing, bioengineering, and pharmaceutical manufacturing. The sample companies are publicly traded in the Shanghai and Shenzhen stock exchanges of China. To obtain a balance panel data, we have excluded the companies which are labeled by ST and ST^*^ (ST or ST^*^ companies’ operating and financial conditions incur changes abnormally, and their stocks may be ceased to trade in China stock exchanges), and deleted companies that have serious missing data. The reason of using balanced panel data follows. According to China’s regulation policies for high-tech listed companies, companies with serious data shortages (unbalanced) generally have bigger operational or managerial problems. That is, these companies might be in an abnormal state of operation. The inclusion of a sample with such companies may biased the results and make the results difficult to be interpreted. Finally, we get 2076 sample observations. Details follow: first, for computer and communication technologies, sample companies:195, observations:1170; for instrument & meter and electronic equipment manufacturing, sample companies:19, observations:114; for bioengineering and pharmaceutical manufacturing, sample companies:132, and observations:792. Our sample data mainly come from the CCER database in China, to ensure the accuracy of the data, we repeatedly collect the same data from the CSMAR database, the result shows that our data has no obvious mistakes.

### Measures

#### Dependent Variable

##### R&D expenditure (RDE)

Observation value is the ratio of R&D expenditures to total sales revenue, R&D expenditures refer to spending money in innovation activities, such as technological improving, new product development, and core technology research.

##### Marketing expenditure (MAE)

Observation value is the ratio of sales expenditures to total sales revenue, which include advertising expenses, sales expenses, business promotion expenses, etc.

#### Independent Variables

##### The proportion of entrepreneurial team with technological background (PTMT)

We define entrepreneurial team with technological background as senior managers with technology-related education, learning and work experience, they may be the members of board of directors, the members of board of supervisors, or other senior managers. The judging criteria are: (1) learning experience of science and engineering in colleges or universities; (2) work experience in the R&D department; (3) work experience in the production department; (4) the work experience in scientific or technological research institutes; and (5) owning the qualification of assistant engineer, engineer, and senior engineer. We calculate the PTMT observation by the ratio of the number of technical executives to the total number of executives, and the number of entrepreneurial team with technological background is collected manually from the resumes of executives of listed companies.

##### The proportion of entrepreneurial team with marketing background (PTMM)

We define entrepreneurial team with marketing background as senior managers with marketing-related education, learning and work experience, they may be the members of board of directors, the members of board of supervisors, or other senior managers. The judging criteria are: (1) learning experience of sales, marketing in colleges or universities; (2) work experience in sale/marketing department; (3) work experience in trading companies, retail stores, and advertising companies, etc. We calculate PTMM by the ratio of the number of marketing entrepreneurial team to the total number of entrepreneurial team, and the number of entrepreneurial team with marketing background is also collected manually from the resumes of executives of listed companies.

#### Control Variables

We control the variables that have the potential to influence R&D and marketing expenditures as follows:

##### Financial performance (ROA)

Firm financial performance has been argued to have a feedback influence on innovation investment, to avoid the endogeneity problem, we use the observation of financial performance in the previous year, and measure it by ROA (calculated by net profit/total assets).

##### Firm size (SIZE)

We measure firm size as the natural logarithm of the total assets.

##### Asset-liability ratio (LEV)

The asset-liability ratio is calculated by total debts/total assets.

##### Cash flow (CASH)

Cash flow is calculated by the cash flow generated in the business activities/total assets.

##### Organizational slack (Slack)

Organizational slack is calculated by the sum of financial expenses and management expenses/sales income.

##### Ownership concentration (TOP1)

Ownership concentration is measured by the share- holding ratio of the largest shareholder.

##### Ownership balance (TOP2)

Ownership balance is measured by the sum of the share-holding ratios of the second to tenth large shareholder.

##### Sales revenue growth rate (growth)

The sales revenue growth rate is calculated by the difference between the current year and the previous year number/ the previous year number.

##### Operating profit rate (profit)

The rate of operating profit is calculated by the formula of total profit/total income.

Other control variables include: Industry, dummy variable.

### Models and Results

#### Regression Equation

To test the above hypotheses, we build the following models (1) and (2): we utilize model (1) to test the effects of top managers with technology and marketing background on R&D expenditure, and use model (2) to examine the influences of top managers with technology and marketing background on marketing expenditure.

(1) RDEi,t=α+0αPTMTi,t1+αPTMMi,t2+αΣkControls+vi+ε+tμi,t

(2) MAEi,t=β+0βPTMTi,t1+βPTMMi,t2+βΣkControls+vi+ε+tμi,t

In model (1) and (2), RDEi,t represents the R&D investment of company i in year t, PTMTi,t represents the proportion otop managers with technological background of company i in year t, PTMMi,t represents the proportion of top managers with marketing-background of company i in year t, and Σcontrols represents all of the control variables. In the equations, α0 and β0 is intercept and αi and β1 is the parameters that need to be estimated. v i and εt represent unobservable individual effects and time effects in the current period, and μi,t indicates the mixed random interference of the individual and time effects.

#### Descriptive Statistics

Table 1 shows the descriptive statistics for main variables, The results indicate that the average proportion of top managers with technical background is 30.5%, the standard deviation is 15.6%, the minimum value is 0, and the maximum value is 0.813, the mean and standard deviation of top managers with marketing background are 11.2 and 9.5%, the minimum and maximum value are 0 and 0.533, which manifest that the proportion of technical executives is higher than that of marketing executives in Chinese listed high–tech companies. In addition, the means of R&D expenditure and marketing expenditure are 6.2 and 12.1%, respectively, and its standard deviations are 7.0 and 12.9%, which indicates that the marketing expenditure of China’s high-tech listed companies is higher than that of R&D on average.

**TABLE 1:** Means, standard deviations (SD), and correlations of variables.

	**Mean**	**SD**	**1**	**2**	**3**	**4**	**5**	**6**	**7**	**8**	**9**	**10**	**11**	**12**
1 RDE	0.06	0.07												
2 MAE	0.12	0.13	0.003											
3 PTMT	0.31	0.16	$0.15^{*,**}$	$-0.07^{*,**}$										
4 PTMM	0.11	0.10	$-0.05^{*}$	$0.12^{*,**}$	$0.13^{*,**}$									
5 ROA	0.05	0.07	$-0.15^{*,**}$	$0.21^{*,**}$	–0.01	0.01								
6 SIZE	21.84	1.04	$-0.09^{*,**}$	$-0.06^{*,*,**}$	$-0.09^{*,**}$	0.01	$0.08^{*,**}$							
7 LEV	0.34	1.91	$-0.14^{*,**}$	$-0.19^{*,**}$	$-0.16^{*,**}$	–0.03	$-0.31^{*,**}$	$0.43^{*,**}$						
8 CASH	0.05	0.07	$-0.07^{*,**}$	$0.13^{*,**}$	0.01	0.05^*^	$0.36^{*,**}$	$0.07^{*,**}$	$-0.19^{*,**}$					
9 TOP1	0.32	0.14	$-0.09^{*,**}$	0.06^∗∗^	–0.01	$0.13^{*,**}$	$0.16^{*,**}$	0.02	$-0.09^{*,**}$	$0.11^{*,**}$				
10 TOP2	0.23	0.12	0.06^∗∗^	0.03	$0.12^{*,**}$	–0.01	$0.07^{*,**}$	$-0.08^{*,**}$	$-0.23^{*,**}$	0.01	$-0.27^{*,**}$			
11 slack	0.27	0.44	$0.17^{*,**}$	$0.26^{*,**}$	–0.04	0.02	–0.01	–0.02	–0.03	0.00	0.01	0.02		
12 profit	0.11	0.37	$-0.28^{*,**}$	$0.07^{*,**}$	–0.03	–0.01	$0.58^{*,**}$	0.01	$-0.24^{*,**}$	$0.16^{*,**}$	0.05^*^	$0.09^{*,**}$	$-0.12^{*,**}$	
13 growth	0.23	0.71	–0.06^∗∗^	0.03	–0.03	0.01	$0.12^{*,**}$	$0.11^{*,**}$	$0.07^{*,**}$	0.00	–0.01	$0.10^{*,**}$	–0.01	$0.08^{*,**}$

^*^P < 0.05, ^∗∗^*P* < 0.01, ^∗∗∗^*P* < 0.001.

Table 1 also indicates that the variation of the study variables is sufficiently large for regression analysis.

#### Correlations Analysis

We used STATA14.0 to analyze the correlation and significance among all variables. Table 2 also reports the correlation coefficients between all variables and the P-value of all correlation coefficients. In Table 2, the correlation coefficient between the proportion of technical top managers and R&D expenditure is 0.15 (*P* < 0.001), and it is consistent with our expectation that their relationship will be positive. The correlation coefficient between the proportion of top managers with marketing-background and R&D expenditure is -0.05 (*P* < 0.05), which shows that the proportion of marketing top managers is negatively related to R&D expenditure on average.

**TABLE 2:** Results of main effect regression analyses.

**Model**	**Model(1)**		**Model (2)**		
**Variables**	**RDE (OLS)**	**RDE (3SLS)**	**MAE (OLS)**	**MAE (3SLS)**	**VIF**
PTMT	0.046^∗∗∗^	0.052^∗∗∗^	-0.037^∗∗^	-0.055^∗∗∗^	1.07
PTMM	-0.034^*^	-0.050^∗∗∗^	0.042^∗∗^	0.108^∗∗∗^	1.05
SIZE	0.003	0.007^∗∗∗^	-0.003	-0.001	1.35
TOP1	-0.039^∗∗^	-0.02	0.016	0.01	1.29
TOP2	-0.0001	0.023	-0.02	-0.017	1.33
growth	-0.002	-0.003	0.004^∗∗^	0.003	1.05
CASH	-0.011	-0.004	0.01	0.06^*^	1.24
Slack	0.14^∗∗∗^	0.159^∗∗∗^	0.143^∗∗∗^	0.413^∗∗∗^	1.20
profit	-0.027^∗∗∗^	-0.044^∗∗∗^	0.010^∗∗^	0.063^∗∗∗^	1.24
LEV	-0.054^∗∗∗^	-0.074^∗∗∗^	-0.073^∗∗∗^	-0.038^∗∗∗^	1.63
ROA (-1)	-0.007	-0.082^∗∗^	0.059^∗∗^	0.252^∗∗∗^	1.38
Industry	-0.034^∗∗∗^	-0.036^∗∗∗^	0.094^∗∗∗^	0.068^∗∗∗^	1.12
_cons	-0.011	-0.101^∗∗^	0.144^∗∗^	0.009	
Observations	1730	1730	1730	1730	
*R*^2^	0.302	0.311	0.438	0.55	
Wald Chi2	423.72^∗∗∗^	781.35^∗∗∗^	409.19^∗∗∗^	2114.18^∗∗∗^	

^*^P < 0.05, ^∗∗^*P* < 0.01, ^∗∗∗^*P* < 0.001.

In addition, the correlation coefficient between the proportion of technical top managers and marketing expenditure is -0.07 (*P* < 0.001), and the correlation coefficient between the proportion of top managers with marketing-background and marketing expenditure is 0.12 (*P* < 0.001), which also support our assumptions that the proportion of top managers with technological background will have negatively influence on marketing expenditure and the proportion of top managers with marketing background will have positively impact on marketing expenditure.

From the data in Table 1, we also find that the correlation coefficients among the independent variables and the control variables are low and that most correlation coefficients are lower than 0.5. The variance inflation factors of all variables are lower than the threshold of 10, which indicates that the multicollinearity problem will not threaten the results of our regression analysis. In order to avoid the influence of multicollinearity on the accuracy of the regression coefficient estimation of independent variables, we also performed VIF test on all independent variables and control variables after regression analysis. The test results are shown in the following Table 2. The regression equation can be seen from the results in the table. The VIF values of all variables in all variables are much less than 10, indicating that there is no obvious multicollinearity problem in the regression equation of this study.

#### Endogeneity Test

If the independent variables are significantly correlated with the regression residual, it will lead to parameter estimation error, we follow the endogeneity test method of Wooldridge (2012), and construct model (3) to test the endogeneity of the main independent variables.

(3) Y=itα+βX+itγZ+it+1φF+itv+iεit

In model (3), Y is the dependent variable, X is the independent variables and control variables, Z is the observations of independent variables and control variables at the t+1 year, F is the time dummy variables, and Vi is the non-observable effect. If all of the coefficients of Z is not significant, the assumption that all variables are exogenous is accepted. If there is a coefficient of Z that is significant, the corresponding variable is endogenous. The regression result of model (3) shows that ROA is an endogenous variable. To eliminate the threat of endogeneity to our parameter estimation, we use the ROA observations at the t–1 year in our regression equations.

#### Regression Analysis

To test the four hypotheses in this study, we have established models (1) and (2) to estimate the regression parameters by the method of single-equation or multi-equation. To compare the difference between the two regression methods, we use single-equation OLS regression and multi-equation 3SLS regression to estimate all the parameters respectively, and the results are shown in Table 2.

Model (1) in Table 2 is utilized to test hypotheses 1 and 4, which predict that top managers with technical background will have a positive effect on R&D expenditure, and top managers with marketing background will have a negative impact on R&D expenditure. We utilize R&D expenditure as the dependent variable, the proportion of top managers with technical background and marketing background are taken as independent variables, we also control main variables that may affect R&D expenditure. To avoid the threats of the endogenous problem to the regression result, the ROA data are delayed by one year, thus, we miss 346 observations and the total number of observations is 1730. The result of single-equation regression shows that the coefficient for the proportion of top managers with technical background (PTMT) is positive and significant (α1 = 0.046, *p* < 0.001), and the 3SLS result indicates that the coefficient for the proportion of top managers with technical background is also positive and significant (α1 = 0.052, *p* < 0.001), both the two results strongly support our hypothesis 1. We argue that top managers with technical background have higher collective psychological capital when dealing with technology-related decision tasks that they are familiar with, because they know how to make R&D investment successful and how to avoid the risk of failure, thus, which makes it easier to produce a psychological state of confidence, optimism and hope. The positive psychological state of technical executives for R&D promotes the increase of R&D expenditure, the higher the proportion of technical executives, the more obvious this positive collective psychological state is and the higher the R&D expenditure is. Our empirical results support these assumptions.

In Table 2, the OLS result shows that the proportion of top managers with marketing background has negative influence on R&D expenditure (α2 = −0.034, *p* < 0.1), and the 3SLS result also indicates that the coefficient for the proportion of top managers with marketing background is negative and significant (α2 = −0.050, *p* < 0.01), which support our hypothesis 4. As mentioned above, because marketing executives are not familiar with the technical field, it is difficult for them to generate positive psychological states such as confidence, optimism and hope for R&D activities, thus, the higher the proportion of marketing executives, the greater the impact of this negative psychological state, and the smaller the R&D expenditure may be. The results of both OSL and 3SLS regression support this hypothesis.

Model (2) in Table 2 is utilized to test hypotheses 2 and 3. We utilize marketing expenditure as the dependent variable, and use the proportion of top managers with technical background and marketing background as independent variables, we also control main variables that may influence marketing expenditure. The result of single-equation regression shows that the coefficient for the proportion of top managers with technical background is negative and significant (β1 = −0.037, *p* < 0.05), and the 3SLS result indicates that the coefficient for the proportion of top managers with technical background is also negative and significant (β1 = −0.055, *p* < 0.01), both the two results strongly support our hypothesis 2. Because technical top managers are relatively short of market-related knowledge and information, they may not be able to accurately understand and judge the significance and value of marketing activities, thus, they may have lower psychological capital for marketing activities. When the proportion of technical executives is higher, the lower TMT’s collective psychological capital for marketing activities, the lower the company’s marketing expenditure may be. Our empirical results fully support this assumption.

In addition, the OLS result in model 2 shows that the proportion of top managers with marketing background has positive influence on marketing expenditure (β2 = 0.042, *p* < 0.05), and the 3SLS result also indicates that the coefficient for the proportion of top managers with marketing background is positive and significant (β2 = 0.108, *p* < 0.01), which strongly support our hypothesis 3 which predict that marketing top managers will be positively related to marketing expenditure. Because marketing top managers have rich market-related knowledge, information and practical experience, they can accurately understand and judge the significance and value of a marketing activity, and know how to overcome difficulties and achieve success. Therefore, top managers with marketing background are prone to positive psychological states such as confidence, optimism, and hope for marketing activities. When the proportion of marketing top managers is higher, TMT’s collective psychological capital for marketing activities is higher, and the company’s marketing expenditure may be higher. The above empirical results provide strong support for this assumption. An additional analysis is conducted thanks to one of the reviewers’ suggestion. We analyzed consider the impact of the interaction between the two on entrepreneurship orientation and market orientation, that is, the interaction of the proportion of team composition. See Table 3 for the results. It can be seen from Table 3 that the regression coefficient of the interaction term in the regression result of Model 1 is significant (α3 = −2.47, *P* < 0.014, indicating that the ratio of marketing background to the technical background positively affects R&D investment. That is, the higher the proportion of senior executives with marketing background, the weaker the positive influence of the technical background executives on R&D investment. Based on the results, we could see that the interaction term negatively influenced the outcome variable. Such result, to some degree (though preliminarily), echoes our central thesis that the inconformity of the influence powers of technical, or marketing dominant teams (that is the influence of technical/marketing proportions is a either-or phenomenon).

**TABLE 3:** Results of interaction regression analyses.

**Model**	**Model 3**	**Model 4**
**Variables**	**RDE**	**MAE**
PTMT	$0.075^{*,**}$	$-0.055^{*,**}$
PTMM	0.050	–0.011
PTMT × PTMM	–0.282^∗∗^	0.183
SIZE	0.004^*^	–0.003
TOP1	–0.041^∗∗^	0.017
TOP2	–0.002	–0.019
Growth	–0.002	0.004^∗∗^
CASH	–0.008	0.009
Slack	$0.140^{*,**}$	$0.143^{*,**}$
Profit	$-0.027^{*,**}$	0.010^∗∗^
LEV	$-0.055^{*,**}$	$-0.072^{*,**}$
ROA (-1)	–0.010	0.061^∗∗^
Industry	$-0.034^{*,**}$	$0.093^{*,**}$
_cons	–0.023	$0.152^{*,**}$
Observations	1730	1730
*R*^2^	0.303	0.441
Wald Chi2	$430.39^{*,**}$	$410.93^{*,**}$

^*^P < 0.05, ^∗∗^*P* < 0.01, ^∗∗∗^*P*<0.001.

## Discussion and Implications

To strengthen the linkage between our practical findings and the extant theoretical bases in the literature, we just adopted the “perspective” of psychological capital to make significant interpretation of our results. Drawing on psychological capital theory, this study analyzes the influence of the professional-background structure of entrepreneurial team on the company’s strategic investment decision. The core proposition is that entrepreneurial team with different background will have different positive psychological capital when they are involved into various decision tasks. Entrepreneurial team with technical background will have higher positive psychological capital than other entrepreneurial team when they face R&D investment decision, thus, the proportion of technical entrepreneurial team will positively influence a company’s R&D investment. On the contrary, technical entrepreneurial team may have low psychological capital when they deal with marketing investment decision, thus, the proportion of technical entrepreneurial team may have negative impact on marketing expenditure. Our empirical results support these posits.

In addition, consistent with theoretical analysis, our empirical results find that the proportion of entrepreneurial team with marketing background has a negative impact on the company’s R&D spending, and has a positive effect on marketing spending. Our study indicates that not only the individual background characteristics of entrepreneurial team will affect the company’s strategic decisions, but more importantly, their group structure will also directly affect the company’s strategic decision results. Our research results further illustrate that psychological capital is a state variable, which may vary with individual or task differences. At the group level, the group’s collective psychological capital will change with the difference of its background structure and decision tasks.

Theoretically and practically, we believe that such results could implicate organizational actors, especially top managers themselves, to know better the collective affective scheme for their strategic preferences.

### Theoretical Implications

Previous empirical studies have broadly examined the relationship between TMT characteristics and strategic decisions. For example, Chaganti and Sambharya (1987) examined the effects of TMT characteristics on innovation and found that firms with TMTs having more production/R&D experience tend to pursue a product innovation strategy (Matsuno et al., 2002). Unfortunately, their study is only based on three firms. Bantel and Jackson (1989) tested showed that TMTs with more education and diverse expertise increase banks innovation degrees. Thomas et al. (1991) found that CEOs with R&D functional backgrounds were preferable to follow an innovation strategy. Barker and Mueller (2002) found that CEOs with science-related degrees have a positive influence on R&D spending. Dalziel et al. (2011) found that the technical experience of outside directors have a positive influence on the R&D spending. Those existing research mainly analyzes the influence of a single characteristic of the entrepreneurial team on some certain strategic decisions, whereas our study contributed by analyzing the influence of different background characteristics of an entrepreneurial team on different strategic decisions and incorporated them into a unified research framework. In addition, our study has examined the effects of the entrepreneurial team with a different background on strategic investment decisions from the perspective of total senior managers, which include the members of the board of directors and board of supervisors, the outside directors, and another entrepreneurial team. Therefore, our study contributes to the literature by extending the scope and content of the above research topic.

On the other hand, our study also contributes to the psychological capital literature by providing an application case. The existing studies find that working environment, leadership behavior, and supervisor support, etc. influence individual psychological capital, and individual psychological capital has an important impact on attitudes, work performance, job satisfaction, and organizational citizenship behaviors, etc. Our research results enlighten that individual background characteristics and task characteristics may also be important factors affecting psychological capital, and this effect can be extended to the collective and organizational levels. Our study also further reveals that the collective psychological capital of the senior management group may affect the strategic decision at the organizational level. Therefore, our study provides a new perspective for expanding the research scope of antecedents and outcomes of psychological capital.

### Managerial Implications

Our research can also provide some enlightenment for corporate executive structure governance. and strategic decision practice in the transitioning countries. Our empirical results showed that entrepreneurial team with different backgrounds often have different decision preferences, and the different background structures of entrepreneurial team will lead to different strategic decisions. Therefore, to improve the quality of their strategic decisions, the company should pay attention to the balance of the senior management structure, when there are too many entrepreneurial members with technical background, the company should increase the number of entrepreneurial members with marketing background to weaken the decision influence of technical members, and vice versa.

In addition, our study also suggests that entrepreneurial team’s collective psychological capital may influence company’s strategic investment decisions, thus, the company can design the effective rules of interaction among entrepreneurial team, which can affect the team’s collective psychological capital in the process of strategic decision-making, thus promoting the team’s work performance, and improving its decision-making quality.

### Limitations and Future Research Directions

Subject to the availability of data, our samples are mainly from Chinese high-tech listed companies, thus, whether our research conclusion also applies to unlisted high-tech companies remains to be tested by future research. On the other hand, although we theoretically analyzed how the structure of entrepreneurial team affect their collective psychological capital, and ultimately affecting investment decisions. Limited by the availability of data, we have not been able to directly test the role of collective psychological capital. Future research can do further work on this point. Another issue is that we have not dealt with managers with both technical and marketing backgrounds, which posed an interesting research topic for the future. Additionally, this article uses the panel data of Chinese listed companies, and the research findings may not apply to foreign companies. Moreover, we only examined the influence of top management team with entrepreneurship orientation and market orientation. Future studies could make similar inquiry on board of directors as an equally important group. Last but not least, while we used the single-dimensioned R&D to measure entrepreneurial orientation, future studies may want to expand such measurement to a multidimensional one (see Li et al., 2008; Williams and Lee, 2009 for the construct’s detailed discussions).

## Data Availability

The datasets generated for this study are available on request to the corresponding author.

## Author Contributions

LG was the major author for the first draft of the manuscript and responsible for the development of propositions. Y-FC facilitated the data analyses. K-HL reviewed and edited the manuscript. C-FL was responsible for the R&R and communication procedures.

## Conflict of Interest Statement

The authors declare that the research was conducted in the absence of any commercial or financial relationships that could be construed as a potential conflict of interest.
